# Recognition of Basic Emotions with and without the Use of Emotional Vocabulary by Adolescents with Down Syndrome

**DOI:** 10.3390/bs12060167

**Published:** 2022-05-30

**Authors:** Régis Pochon, Claire Touchet, Laure Ibernon

**Affiliations:** 1 Cognition, Health and Socialization Laboratory, University of Reims Champagne-Ardenne, 51097 Reims, France; 2 Research Center in Psychology, Cognition, Psyche and Organizations, University of Picardie Jules Verne, 80025 Amiens, France; claire.touchet@me.com (C.T.); laure.ibernon@u-picardie.fr (L.I.)

**Keywords:** emotional vocabulary, emotion recognition, down syndrome, emotional competence

## Abstract

Background: Children with Down syndrome (DS) often experience behavioral and emotional issues that complicate their socialization process and may lead to psychopathological disorders. These problems may be related to deficits affecting emotional knowledge, particularly emotional vocabulary. Because emotional vocabulary makes it easier for typically developing children to identify emotions, a deficit affecting it in DS could be problematic. Methods: Twenty-eight adolescents with DS matched with typically developing (TD) children for their score on the Benton Facial Recognition Test were asked to recognize six emotional expressions presented in the form of filmed sequences, based on (1) nonverbal cues such as prosody, and (2) an emotional label. Results: The adolescents with DS recognized the six basic emotional expressions at a level comparable to that of the TD children in both conditions (with and without emotional vocabulary), but the facilitating effect of vocabulary was lower in that group. Conclusions: This study does not show a deficit affecting emotion recognition in DS, but it emphasizes the importance of early acquisition of emotional knowledge in this syndrome. Regular and varied use of internal state words should be encouraged in familial interactions, and education should include specifically adapted social and emotional learning programs.

## 1. Introduction

As Iarocci et al. [1] pointed out, socially appropriate behavior requires the implementation and coordination of cognitive and emotional processes to produce the necessary responses in different contexts. Improved knowledge of such processing capacities in people with Down syndrome (DS) is important for a better understanding and improved treatment of the problems they encounter in social interactions, especially when those interactions become more complex during their education [2,3,4]. At preschool age, despite the positive stereotype describing children with DS as warm and highly sociable [5], their social development can be more complicated than that of typically developing (TD) children for various reasons. After questioning the mothers of children with DS aged 54 to 83 months, Guralnick et al. [6] found that those children had a less extensive social network of peers and poorer dyadic interaction skills than TD children of the same developmental age. Furthermore, Guralnick et al. [7] directly observed children with DS of the same age as in their previous study in a dyadic play situation: although several aspects of their interactions with their TD peers were as expected for their developmental level, the children with DS had more difficulties maintaining sustained interactive play. 

Later, at school age, children with DS may manifest more undesirable behaviors (i.e., stubbornness, oppositionality or inattention) and emotional problems (i.e., emotion regulation) than their TD peers [8,9,10,11,12] and these undesirable behaviors have negative consequences for their adaptation to school [13,14]. Nevertheless, it is important to note that they show fewer inappropriate (maladaptive) behaviors than other students with developmental disabilities. At the end of adolescence, many people with DS still present behavioral problems with externalized symptoms [15], but less than in the case of young people with intellectual disability without Down syndrome [16]. Internalized symptoms such as depressive symptoms and isolation tendencies appear more often [8,16].

In view of the behavioral and emotional issues observed in other interactive contexts that can be as crucial as schooling, it appears essential to continue studying the development of emotional knowledge in people with DS. This is a very important question because the development of social competency depends on a certain number of sociocognitive resources, among which the ability to efficiently process emotional information plays a crucial role (see Trentacosta and Fine, [17], for a review). Of the varieties of emotional knowledge, this study’s specific objective is to study the role of emotional vocabulary in recognizing the basic emotions (happiness, sadness, anger, fear, surprise, and disgust) in adolescents, an age group that is under-represented in behavioral studies of DS [18]. According to Saarni [19,20], mastery of the vocabulary associated with emotions and the ability to recognize other people’s emotions are key components of emotional competence. The words referring to emotions allow somebody to share their emotional experience and efficiently represent the emotions felt by other people. In Saarni’s view, the development of emotional competence is inseparable from the development of social competence.

The role of emotional vocabulary in the recognition and understanding of emotions has been highlighted by several studies of TD children. Russell and Widen [21,22] showed that the use of words resulted in a better understanding of the causes of fear and disgust than the presentation of facial expressions; they found a label superiority effect in the recognition of happiness, anger and fear. In a later study of four-year-old children [23], the emotional label proved to be a stronger cue than facial expression in describing the causes and behavioral consequences of an emotion. Similarly, emotional terminology enhanced the speed and accuracy of young adults’ emotion perception in the study by Lindquist et al. [24]. This research led Salmon et al. [25] to highlight how important it is for the development of emotional knowledge—including recognition of emotions and understanding of their causes—to train children to label emotions. Finally, the development of emotional vocabulary was studied by Declercq et al. [26] in children aged four to seven years old. Their results showed that concrete words are acquired earlier than emotion words or abstract words, but emotion words come earlier than abstract words. This result confirms that this class of words differs from abstract words from a developmental perspective. Because it has been clearly shown that a well-developed emotional vocabulary fosters the recognition and understanding of emotions in children and young adults, it is very important to consider this factor in the study of emotional knowledge in people with DS, who have considerable language problems although their receptive vocabulary is relatively preserved [27,28,29].

Most recent studies of emotional expression recognition in DS have used tasks that mobilize emotional vocabulary and have shown deficits in both children [30,31,32,33,34] and adults with DS [35,36]. It is important to note that the deficits found in these studies are rarely general but tend to concern certain specific expressions. The expressions that have often proven problematic for participants with DS have mainly been expressions of fear [32,33,37], surprise [34], anger [30] and sadness [31]. However, in a longitudinal study, Pochon and Declercq [38] found no differences in emotion recognition between children with DS (6.0–14.8 years), children with nonspecific intellectual disability (NSID) and TD children. Recognition was assessed with a nonlexical task that involved matching an emotional vocalization that was heard with an expressive face presented in a photograph. The results led the authors to hypothesize that the use of emotional vocabulary in earlier emotion recognition studies may have disadvantaged people with DS [39].

This hypothesis was contradicted by the results of Channell et al. [40], who did not find a recognition deficit in children with DS (6.7–18.4 years) who had to watch a short video sequence that presented a character expressing an emotion and then had to choose an emotional term from among three orally presented ones (happy, sad or scared). In the study by Pochon et al. [41], adolescents with DS (10.5–18.9 years) succeeded as well as their TD peers of the same developmental age (DA) when the recognition of the six basic emotional expressions involved using dynamic stimuli (video sequences) and not emotional vocabulary. Finally, Cebula et al. [37] specifically studied the impact of emotional labels on the recognition of emotions by adolescents with DS (10.9–18.7 years) and NSID and by a TD group of the same DA. In all three groups, the use of an emotional label allowed for more accurate responses than the use of photos of expressive faces. Nevertheless, although the DS group performed as well as the other groups overall, these participants failed more often than the TD group at recognizing fear, whether or not the word fear was uttered.

As Cebula et al. [42] pointed out, although the hypothesis that there is a specific deficit affecting emotion recognition in DS is corroborated by most studies, there is not enough evidence to support it, and it generally involves subtle differences rather than obvious deficits such as those observed in autistic spectrum disorders. Nevertheless, given the existence of other subtle differences in other socio-emotional developmental domains such as social referencing, joint attention and theory of mind (see Cebula et al., [42]; Fidler and Nadel, [4], for a review), the cumulative developmental differences could have a negative impact on the ability of young people with DS to efficiently process emotional information. The original feature of this study is that it directly compares the recognition of emotional facial expressions, depending on whether or not recognition is solicited with emotional vocabulary, and with dynamic stimuli (video sequences), as recommended by Moore [43], to ensure better ecological validity. In addition, control conditions of non-emotional recognition were introduced in order to differentiate between emotional knowledge and other cognitive skills, as suggested by Kasari et al. [30] and Moore [43].

A dynamic task for emotional facial expression recognition was used in this study, which included two main conditions. In the first, “nonlabel,” condition, no emotional terms were used to stimulate recognition, which was based on nonverbal cues. In the second, “label,” condition, participants were asked to describe an expression after hearing an emotional label.

In view of the conclusions of earlier studies, we expected to find (1) a higher level of recognition of basic emotional expressions when an emotional label is used [21] but with a lesser effect on adolescents with DS than on a TD group [39]; and (2) more difficulty for adolescents with DS than for TD adolescents in recognizing fear based on an emotional label [37], but comparable results for fear when recognition is based on nonverbal cues [41].

## 2. Method

### 2.1. Participants

Twenty-eight French adolescents with Down syndrome aged from 126 to 240 months old participated in this experiment (15 boys, 13 girls). Three of them had attended regular school with follow-up by a specialized service and the others were in medical and social institutions. The diagnosis of trisomy 21 was confirmed by the medical teams of the institutions and services treating the young people. To participate in the study, these adolescents with DS had to be able to correctly understand the instructions given during the preliminary examinations described below. Adolescents with sensory deficits, major attention disorders or autistic spectrum disorders were excluded. These participants were matched with 28 typically developing children aged 42 to 65 months (17 boys, 11 girls) recruited in daycare centers or elementary schools. None of them had any sensory, physical or mental disorders or any significant lag in their schooling and all were monolingual French speakers. The matching was done individually based on the score on the Benton Facial Recognition Test (BFRT; [44]). This is a standardized test that assesses the ability to identify unfamiliar faces. The adolescents with DS performed at the same level as younger children on this preliminary measurement. As expected, there was no difference between the matched groups (*t*(54) = −0.19, *p* = 0.85). Nonverbal reasoning was assessed with the general score on Raven’s Colored Progressive Matrices (RCPM; [45]); the means for the two groups did not differ significantly (*t*(54) = 1.18, *p* = 0.24). Finally, the participants with DS took the block design subtest of the French version of the Wechsler Preschool and Primary Scale of Intelligence (WPPSI-III; [46]), which provides an estimate of their nonverbal DA. The characteristics of each group are presented in Table 1.

### 2.2. Ethical Approval

Ethical approval was not required for this study, according to the national and institutional guidelines. This study was carried out in accordance with the recommendations of French law that written informed consent be obtained from all subjects. All participants gave their oral consent to take part in the study. The informed consent of their parents and/or legal guardians was obtained in writing after they had been informed of the goals of the study, the kinds of tests that would be administered, and the fact that they could withdraw their consent at any time. Their informed consent was received in writing in accordance with the Declaration of Helsinki. The academic and medical–social authorities were also informed and gave consent for the students to take part, because the meetings usually took place at the educational institution.

### 2.3. Task and Procedure

The primary objective of this experiment was to compare basic emotion recognition depending on whether emotional vocabulary was or was not used in recognition. To do this, we used a task that comprised two conditions, the design and presentation of which were identical but which prompted recognition in different ways: (1) a “nonlabel” condition where recognition involved associating the intonation and emotional timbre of a sentence that was heard with a facial expression; and (2) a “label” condition in which recognition involved associating an emotional label with a facial expression. Each of these conditions had a related non-emotional control condition, which meant that there were four conditions in total (two emotional conditions and two non-emotional control conditions). The use of control conditions with the same level of cognitive demand as the experimental conditions allowed us to be sure that the instructions were understood and that any difficulties were not due to the task itself but to a deficit affecting emotional information processing. Responses time were not recorded. The task administered was based on the nonverbal task presented in the study by Pochon et al. [41]; thus, only the main characteristics of the task will be presented here.

#### 2.3.1. Nonlabel Conditions

- Nonlabel non-emotional (control)

Six familiar objects were used in this condition: a small plastic bottle, a ceramic bowl, a metal cooking pot, a stemmed glass, a plastic citrus juicer, and a plastic kitchen spatula. Each one appeared as a target three times. They were presented in short video sequences (3.2 s), in which each object was struck by another object—9 times by a large wooden spoon and 9 times by a large metal spoon. The objects were hit in three different ways: three blows, two double blows and three double blows. Each method was used six times. For each presentation, two video sequences presented simultaneously showed the same hitting object and the same hitting method; only the object that was hit differed (a target object and a distractor, for example the bowl and the bottle). The video sequences were shown side by side with a small space between them and were displayed simultaneously after presentation of a target encouraging the participant to look at the center of the screen. The experimenter then asked the participant to indicate which video sequence corresponded to what had been heard on the soundtrack. The video sequence was played in a loop until the participant answered; the participants responded by manually pointing at the screen of a portable computer (15″, resolution of 1366 × 768 pixels). The maximum score on this task was 18 (6 target objects presented 3 times).

- Nonlabel emotional

This condition involved the presentation of six emotions—happiness, sadness, anger, disgust, surprise and fear—in the form of video sequences, which were shown in the exact same way as in the control task. Each emotion was the target emotion three times. They were expressed by two professional actors (9 times by a man, 9 times by a woman) who were only visible from the shoulders up. These two actors were specially trained to express basic emotions with their face and voice, and a substantial number of takes were necessary. The selected video sequences were correctly identified in 95% of cases by 20 non-expert adults aged 20 to 40 years. The actors spoke three sentences in alternation: two were in French (Léa est venue en avion “Léa came by plane”; La bouteille est sur la table “The bottle is on the table”), and one was made up of nonwords (Cognogo tiketou). These sentences, which did not contain any emotional information, were spoken by the actors with the target intonation and facial expression. Each one was used six times. In this emotional condition, two video sequences were played with a single soundtrack, which corresponded to the target emotion. This soundtrack was played with a time lag (desynchronization) so that the synchronization of the sound with the actor’s lip movements could not be used as a clue. For example, the computer simultaneously played two video clips showing the same actor, with an angry face on the left side of the screen and a fearful face on the right side of the screen. On each video, the actor spoke the same sentence; during the presentation, the participant heard only the voice corresponding to the target emotion, with a time lag. To answer correctly, participants had to rely on the intonation and vocal quality of the sentence they heard to identify the correct facial expression from the two filmed sequences. The maximum score on this task was 18 (6 target emotions presented 3 times).

#### 2.3.2. Label Conditions

For the label conditions, the visual material and presentation conditions described above were essentially reused, as were the number and order of the items. The only difference was the fact that the soundtrack for all the video sequences was eliminated and participants were asked to answer a question concerning a characteristic of the objects in the non-emotional condition or an emotional label in the emotional condition. For each condition of this task, participants had to answer by pointing directly at the screen, and the maximum score was 18 (6 target objects or target emotions presented 3 times).

- Label non-emotional (control)

The video sequences presenting the hit objects were played but they were silent, without a soundtrack. When the videos started, the experimenter provided instructions such as “Show me which video shows an object that is fragile and transparent” or “Show me which video shows an object that is used at breakfast”.

- Label emotional

The filmed facial expressions were shown but the voice was not heard, because the soundtrack was eliminated so that recognition relied only on the facial expression. When the videos started, the experimenter provided instructions such as “Look carefully at the two videos and show me the one where the lady is happy” or “Look carefully at the two videos and show me the one where the man is surprised”.

The meetings with participants took place in a quiet, familiar room in each participant’s educational institution. All the participants were met during their ordinary educational activity hours. To ensure that the results would not be affected by fatigue, boredom or concentration problems, the preliminary tests and experimental tasks were administered in three sessions lasting 20 to 30 minutes each. The nonlabel conditions were always administered before the label conditions. Thus, in the nonlabel emotional condition, participants watched the videos without having seen them associated with an emotional label, which could have constituted a clue for recognition. The nonlabel conditions were presented in 3 blocks of 12 items: 6 items in the non-emotional condition and 6 items in the emotional condition presented alternately within each of the 3 blocks. The label conditions were presented in exactly the same way. Because of the disorientation due to the lag between sound and image in the nonlabel conditions, they were preceded by an additional 6-item training block that was not included in the final score. These items allowed the participants to become familiar with the task and the experimenter to be sure that they understood the instructions. To refocus attention and maintain motivation, there was a break after each block, during which the experimenter chatted with the participant. The initial instructions were as follows: “Listen carefully to me. When I press this button, you’ll see two short films, one on the left and one on the right. [The experimenter showed the locations on the blank screen.] When those short films start, I’m going to ask you a question about them. Now, we’re starting. Are you ready? Watch this target closely”.

## 3. Results

Because the mean scores obtained by the two groups in the various conditions had to be compared with parametric tests, the normality of the distributions for each variable studied was tested in advance for each group using one-sample Kolmogorov–Smirnov tests. The distribution was normal for the overall results in the various conditions, which made it possible to perform an analysis of variance (ANOVA) followed by a post hoc test (Tukey’s test). These analyses were followed by a correlational study to identify relations between success on the preliminary tests and success on the experimental tasks.

### 3.1. Accuracy Data Analyses

In the task, participants had to match a short video sequence (non-emotional or emotional) with an auditory or auditory–verbal stimulus (nonlabel or label). Two dependent variables in both conditions were taken into account: Label (nonlabel, label) and Emotion (non-emotional, emotional). The cross-tabulation of these two variables gave rise to four conditions: nonlabel non-emotional, nonlabel emotional, label non-emotional and label emotional.

A 2 × 2 × 2 (Group, Label, Emotion) mixed-design ANOVA was used to compare the scores obtained by participants (Table 2). The analysis revealed no significant main effect of Group (*F*(1, 54) = 1.408, *p =* 0.240, *η_p_*^2^ = 0.025), but there was a significant main effect of Label (*F*(1, 54) = 52.06, *p* < 0.001, *η_p_*^2^ = 0.490), indicating better performance in label than in nonlabel conditions. However, this label effect was qualified by a significant Group × Label interaction (*F*(1, 54) = 10.05, *p =* 0.002, *η_p_*^2^ = 0.157). The main effect of emotion was also significant (*F*(1, 54) = 58.82, *p* < 0.001, *η_p_*^2^ = 0.521), with higher scores in the non-emotional conditions than the emotional conditions; the effect of the Group × Emotion interaction was not significant, nor was the second-order Group × Label × Emotion interaction.

The post hoc analyses of the Group × Label interaction showed that the TD group performed better than the DS group in the label conditions (+1.45, *p* = 0.042). Both groups had better results in the label than in the nonlabel conditions; the difference was greater for the TD group (+3.1, *p* < 0.001) than for the DS group (+1.2, *p =* 0.03).

The post hoc analyses of the second-order Group × Label × Emotion interaction revealed no intergroup differences, but there was a significant effect of label in the non-emotional (+3.11) and emotional (+3.04) conditions for the TD group (*p* < 0.001). For the DS group, the effect of label was not significant in the non-emotional condition (+0.82, *p =* 0.592) but was significant for the emotional condition (+1.57, *p =* 0.018). In both groups, emotion recognition was better with a label than without one.

### 3.2. Analysis of Response Accuracy According to Emotional Expression

**Figure 1:**
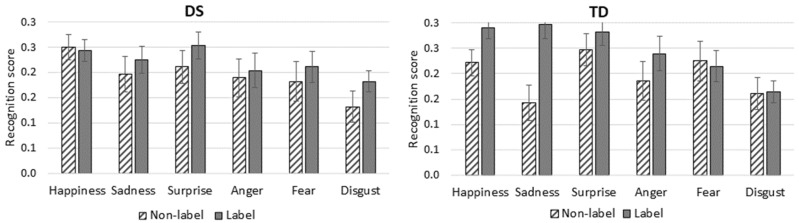
Mean recognition score (95% C.I.) for Each Emotional Expression by Group and Condition (max. score = 3).

To determine whether the groups differed in their recognition of the six emotional facial expressions they were shown (Figure 1), recognition scores for each expression were analyzed with a 2 × 2 × 6 (Group × Label × Expression) mixed-design ANOVA. The main effect of group was not significant, although it approached significance (*F*(1, 54) = 3.589, *p =* 0.064, *η_p_*^2^ = 0.062), but there was a main effect of label (*F*(1, 54) = 37.68, *p* < 0.001, *η_p_*^2^ = 0.411) and of Expression (*F*(5, 270) = 19.19, *p* < 0.001, *η_p_*^2^ = 0.262). The Group × Label interaction was not significant, although it approached significance (*F*(1, 54) = 3.807, *p =* 0.056, *η_p_*^2^ = 0.065), whereas the Label × Expression interaction was significant (*F*(5, 270) = 3.90, *p =* 0.002, *η_p_*^2^ = 0.067). However, these effects were qualified by a significant second-order Group × Label × Expression interaction (*F*(5, 270) = 5.79, *p* < 0.001, *η_p_*^2^ = 0.097).

The post hoc analyses of the second-order Group × Label × Expression interaction did not reveal any intergroup difference regardless of expression or of whether or not a label was used. For the TD group, the expression of sadness was better recognized in the label than in the nonlabel condition (*p* < 0.001), whereas no intragroup difference was found for the DS group.

### 3.3. Correlations between Preliminary Test Results and Scores in Emotional Conditions

For each group, correlation coefficients were calculated for each measure characterizing the group and the results in both emotional conditions of the experimental task (Table 3). In the adolescents with DS, the coefficients were generally low; only one coefficient was higher than 0.20. The correlation between RCPM score and recognition of expressions in the label condition was significant with a large effect size, but no such correlation was found in the non-label condition. In addition, emotion recognition performance was not correlated with chronological age or BFRT score regardless of condition, with or without a label. Among TD children, the correlation coefficients ranged from 0.35 to 0.47 and were significant or approached significance. Chronological age, RCPM score and BFRT score were correlated with emotion recognition in every condition, with a medium to large effect size.

## 4. Discussion

The objective of this study was to study the role of emotional vocabulary in the recognition of emotional facial expressions by adolescents with Down syndrome in comparison with typically developing children. Their emotion recognition performance was analyzed according to whether recognition was or was not prompted by emotional vocabulary. Overall, the results suggest that it is essential to promote the acquisition of an extensive vocabulary of emotion words by children and adolescents with DS to enhance their chances of becoming emotionally competent in their daily social interactions.

The two groups (DS and TD), which were matched for their ability to identify faces (BFRT; [44]), had to recognize six basic emotional expressions, first based on the intonation of a sentence without any emotional significance and then based on an emotional label mentioned in the instructions; two non-emotional control conditions (with or without vocabulary-based prompting) were also administered. Overall—that is, in both emotional and control conditions—the adolescents with DS performed worse than the TD group when lexical knowledge was called upon, whereas there was no intergroup difference when lexical knowledge was not required. Nevertheless, the performance of the adolescents with DS did improve significantly because of their lexical knowledge but less than that of the TD group. More specifically, this improvement was essentially due to better responses in the emotional conditions, whereas vocabulary-based prompting did not result in better responses in the non-emotional conditions. Among the TD children, the use of relevant vocabulary led to better responses in both emotional and non-emotional conditions. To sum up, the results showed that, for all emotions, in both groups, the use of relevant vocabulary allowed for better emotion recognition than nonverbal clues, but that the increase was less marked in the adolescents with DS. These results confirm our first hypothesis. Finally, our analysis of recognition performance for the different emotions confirmed that results improved with vocabulary-based prompting but did not show that either group performed better than the other, regardless of the emotion. Our hypothesis that adolescents with DS would find it more difficult to recognize fear, on the basis of the label, was not confirmed by these analyses.

First of all, it is important to point out that this study did not reveal any significant difference in emotion recognition between the two groups, whether recognition depended on prosodic factors or on an emotional label. This lack of an emotion recognition deficit in participants with DS, of the same DA as the TD group, corroborated the study by Channell et al. [40], which also made use of dynamic stimuli (video sequences), and the study by Pochon and Declercq [38], which made use of vocalizations and static stimuli (photos). However, these results are contrary to those of most studies of emotion recognition in children with DS [30,32,33,34,37,39]. This difference in results, with better performance by people with DS, might be explained by the choice of different methodologies, particularly the nature of the stimuli presented to participants: video sequences instead of photos, as in the study by Channell et al. [40], versus the use of auditory emotional cues (prosody), as in the study by Pochon and Declercq [38], in which vocalizations were heard. The use of dynamic visual stimuli might explain why the deficits in people with DS affecting the recognition of certain emotional expressions mentioned in the literature [30,31,33,34], especially fear [32,37], were not observed in this study. The advantage related to the use of video sequences supports the conclusions of Harwood et al. [47], who found better emotion matching with dynamic than static stimuli in people with intellectual disabilities. Nevertheless, other studies are necessary to establish the exact role of these methodological choices in performance on emotional recognition tests.

Although unexpected, the fact that the performance of the adolescents with DS was generally poorer in the conditions requiring lexical knowledge is not surprising, given that language is one of the areas of functioning most affected in DS [48,49,50]. These conditions called upon receptive language, and specifically receptive vocabulary, which is relatively preserved compared with language production, a domain where people with DS tend to have substantial difficulties [27,28,51]. Although receptive vocabulary is known to be a developmental strong point in DS, it is possible that there may have been a general disadvantage, because participants’ lexical level was not controlled for in this study. That said, a significant effect of label was observed in the emotional condition for participants with DS, although the effect was not as strong as in the TD children. This finding is in accordance with the study by Cebula et al. [37] in which the use of labels enhanced the accuracy of responses by children with DS, except for fear, in comparison with a photo-matching task.

The facilitating effect of emotional vocabulary in the recognition and understanding of emotions demonstrated by Russell and Widen [21,22] and Widen and Russell [23] also seemed to appear in the adolescents with DS. This result suggests that the acquisition of emotional vocabulary in DS is in fact part of the development of emotional competence, as it improves the accuracy of emotion recognition [19]. Nevertheless, because the facilitating effect of vocabulary was not as strong in the DS group as in the TD group, more detailed studies of the composition of receptive vocabulary, in which different kinds of words would be compared to words referring to emotions, will be invaluable for verifying that the understanding of emotional vocabulary in people with DS corresponds to their DA.

This study showed that the level of recognition of basic emotions by adolescents with DS was consistent with their level of nonverbal development, whether or not the recognition process involved motional vocabulary. It also showed that, because of their knowledge of words referring to emotions, these adolescents improved their recognition performance, even though they received less of a benefit than the TD children. However, the comparisons were made between two groups where the mean chronological age was quite different, and the greatest life experience of the adolescents with DS was not considered. Still, although this study contributes to a better understanding of emotional development in people with DS, it focused on emotion recognition and emotional vocabulary and does not address the other components of emotional competence, particularly those requiring more in-depth processing of emotional information. The study of emotion comprehension, for example, could reveal differences related to the quality of executive control [52]. Moreover, this study does not provide any information on the relation between our observations in the experiment and the participants’ actual emotional and relational functioning in their daily lives.

This limitation makes it difficult to generalize, especially regarding whether the knowledge expressed in the results can be considered as reflecting actual emotional competence. The cross-tabulation of these results with behavioral information provided by the families and the educational institutions would have shed light on this matter and allowed for more accurate, more generalizable conclusions. Nevertheless, the meta-analysis by Trentacosta et al. [17] on the correlation between emotional knowledge, on one hand, and social competence and behavior problems during childhood and adolescence, on the other, revealed a relatively consistent relation. This result supports the value of our study and encourages us to continue studying emotional knowledge by applying innovative methods, as we have sought to do by administering a task that involved matching video sequences and nonverbal cues such as prosody.

This study highlights the importance of including the development of emotion-related language in the support provided to young people with DS, to encourage not only receptive understanding but also the use of emotional terms in their speech production. Beeghly and Cicchetti [53] showed that children with DS produced fewer words concerning their internal states than their peers of the same DA. This support could be provided in class or in a specialized institution, by adapting social and emotional learning programs such as the RULER Feeling Words Curriculum [54], but it would doubtless be most effective if it is included in early interventions with families. Tingley et al. [55] noted the use of fewer internal state words in the discourse of mothers and their children with DS, and in particular fewer words related to affect. There is no doubt that early work with families to promote the use of words referring to feelings and emotions in daily familial interactions is indispensable. In fact, it is essential because, as Annette Karmiloff-Smith pointed out, growing up with a developmental disorder, whether it is diagnosed at birth or at a more advanced age, fundamentally changes the linguistic environment by changing both the quantity and the quality of interactions [56].

## Figures and Tables

**Table 1:** Characteristics of BFRT-Matched Groups.

Variables	Group				*t* Value
	Down Syndrome		Typically Developing		
	Mean	*SD*	Mean	*SD*	
Chronological age	185.13	33.75	54.54	7.22	20.02 ***
BFRT score	31.07	3.40	31.25	3.53	0.19
RCPM raw score	14.86	4.95	16.57	5.86	1.18
Blocks (WPPSI-III) age equivalent	53.32	14.68	(54.54 ^†^)	(7.22 ^†^)	-

Note. *n* = 28 in each group. Ages are reported in months. *** *p* < 0.001. ^†^ TD children were not given this test. Chronological age is presented for purposes of comparison. BFRT: Benton Facial Recognition Test; RCPM: Raven’s Colored Progressive Matrices; WPPSI-III: Wechsler Preschool and Primary Scale of Intelligence.

**Table 2:** Accuracy Scores for Each Condition of the Experimental Task.

	Group			
	Down Syndrome		Typically Developing	
Conditions of the task	Mean	*SD*	Mean	*SD*
Nonlabel non-emotional	14.29	2.26	13.21	2.53
Nonlabel emotional	11.61	2.02	11.82	2.92
Label non-emotional	15.11	2.59	16.32	1.59
Label emotional	13.18	2.51	14.86	1.71

**Table 3:** Correlations among emotional conditions of Experimental Task Scores and Assessment Measures by BFRT-Matched Group.

	Group			
	Down Syndrome		Typically Developing	
Variables/Conditions	Non-Label Emotional	Label Emotional	Non-Label Emotional	Label Emotional
Chronological age in months	0.034 (0.862)	0.177 (0.367)	**0.374 (0.050)**	0.362 (0.058)
BFRT score	0.096 (0.628)	–0.049 (0.803)	**0.464 (0.013)**	**0.403 (0.033)**
RCPM raw score	0.087 (0.661)	**0.420 (0.026)**	0.350 (0.067)	**0.469 (0.012)**
WPPSI-III Blocks age equivalent	–0.008 (0.968)	0.063 (0.751)	- ^†^	- ^†^

Note. *n* = 28 in each group. Pearson’s correlations (*r*, with *p* value in parentheses). Significant correlations are presented in **bold type**. ^†^ TD children were not given this test. BFRT: Benton Facial Recognition Test; RCPM: Raven’s Colored Progressive Matrices; WPPSI-III: Wechsler Preschool and Primary Scale of Intelligence.
